# Magnetic and Magnetostrictive Properties of Sol–Gel-Synthesized Chromium-Substituted Cobalt Ferrite

**DOI:** 10.3390/gels9110873

**Published:** 2023-11-02

**Authors:** Chandra Sekhar Beera, B. Dhanalakshmi, D. Nirmala Devi, D. Vijayalakshmi, Akanksha Mishra, S. Ramesh, B. Parvatheeswara Rao, P. Shyamala, Melita Menelaou, Nadyah Alanazi, Abdullah N. Alodhayb

**Affiliations:** 1 Vignan’s Institute of Engineering for Women (Autonomous), Visakhapatnam 530046, AP, India; 2 Vignan’s Institute of Information Technology (VIIT-A), Visakhapatnam 530049, AP, India; 3 Department of Physics, GSS, GITAM Deemed to be University, Visakhapatnam 530045, AP, India; 4 Department of Physics, Andhra University, Visakhapatnam 530003, AP, India; 5 Department of Chemistry, Andhra University, Visakhapatnam 530003, AP, India; shyamalapulipaka06@gmail.com; 6 Department of Chemical Engineering, Cyprus University of Technology, 30 Arch. Kyprianos Str., Limassol 3036, Cyprus; melita.menelaou@cut.ac.cy; 7 Department of Physics and Astronomy, College of Science, King Saud University, Riyadh 11451, Saudi Arabia; nalenazi@ksu.edu.sa

**Keywords:** crystallite size, magnetostriction, anisotropy, saturation magnetization, cobalt ferrite

## Abstract

Chromium (Cr)-doped cobalt ferrite nanoparticles were synthesized using a sol–gel autocombustion method, with the chemical formula CoCr_x_Fe_2x_O_4_. The value of x ranged from 0.00 to 0.5 in 0.1 increments. X-ray diffraction analysis confirmed the development of highly crystalline cubic spinel structures for all samples, with an average crystallite size of approximately 40 to 45 nm determined using the Scherrer equation. Pellets were prepared using a traditional ceramic method. The magnetic and magnetostrictive properties of the samples were tested using strain gauge and VSM (vibrating sample magnetometer) techniques. The results of the magnetic and magnetostrictive tests showed that the chromium-substituted cobalt ferrites exhibited higher strain derivative magnitudes than pure cobalt ferrite. These findings indicated that the introduction of chromium into the cobalt ferrite structure led to changes in the material’s magnetic properties. These changes were attributed to anisotropic contributions, resulting from an increased presence of Co^2+^ ions at B-sites due to the chromium substitutions. In summary, this study concluded that introducing chromium into the cobalt ferrite structure caused alterations in the material’s magnetic properties, which were explained by changes in the cationic arrangement within the crystal lattice. This study successfully explained these alterations using magnetization and coercivity data and the probable cationic dispersion.

## 1. Introduction

Ferrites have a diverse set of electromagnetic characteristics that make them valuable for various technical applications. Ferrites typically exhibit higher permeability compared to other materials, along with higher magnetization and resistivity, while showing lower core losses. Ni-Zn- and Mn-Zn-based ferrites are known for their relatively high magnetization properties. This makes them suitable for applications where strong magnetic fields are required, such as automated devices. On the other hand, cobalt-containing ferrites, such as CoFe_2_O_4_, with high magnetization and Curie temperatures, are less attractive for such applications due to their high positive magneto-crystalline anisotropy and negative magnetostriction. These unique features have recently been identified as being better suited for magneto-mechanical stress or strain monitoring and actuation applications [1]. It has been noticed that rare-earth-based iron (Fe) compounds, with their rich magnetostrictive characteristics, have captured the full focus of sensing applications [2,3]. However, due to their high limits of anisotropy and other constraints, such as poor mechanical stability and excessive pricing [4,5], these materials also have low sensitivity to stress and, hence, offer only limited options for sensing.

To overcome these limitations, current research has focused on producing a ferrite-based material with larger magnetostrictive strains considered for lesser magnetic field strengths. Cobalt ferrite (CoFe_2_O_4_) is one of the replacements being studied to increase its magnetic and magnetostrictive properties. As a result, it was discovered that strengthening cobalt ferrite can cause variations in the cation distribution between the octahedral and tetrahedral sites, resulting in desirable magnetic characteristics. It was also demonstrated that changing the doping concentration of various elements can effectively modify the corresponding magnetic parameters, such as the Ms (saturation magnetization), Hc (coercivity), Tc (Curie temperature), and magnetostriction order (λ and dλ/dH) of cobalt ferrite.

The prime purpose of the work reported here is to study the impact of chromium (Cr) ions on the divalent and trivalent sites when they replace Fe ions in cobalt ferrites. For this goal, cobalt-based ferrite systems with varying amounts of chromium ion replacements are produced and studied. In addition, this work also discusses the observed variations in various magnetic and magnetostrictive properties, such as saturation magnetization, magnetostriction, strain derivative, and coercivity, with respect to Cr substitution in the CoCr_x_Fe_2−x_O_4_ samples. The impact of replacements, grounding methods, and the chemical environment on the potential ionic preferences of distinct cations is also thoroughly discussed to better understand the differences. The experimental changes with Co-Cr ferrites discovered here are linked in accordance to the reported results of Co-Mn ferrites during the procedure to better understand the improvements related to Cr replacements rather than Mn substitutions. At the outset of the debate, an acceptable composition with the necessary properties of high strain derivative, high saturation magnetostriction, and feasible saturation magnetization for a good torque sensor is offered [6,7].

## 2. Results and Discussion

### 2.1. XRD Analysis

**Figure 1:**
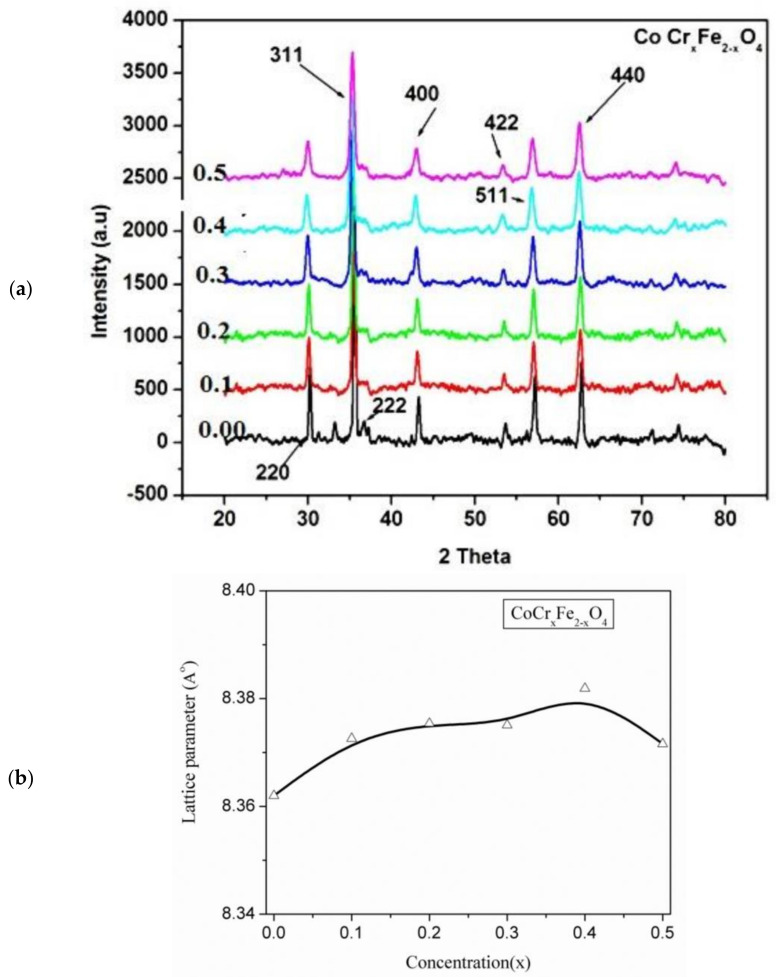
(**a**) XRD patterns of CoCr_x_Fe_2−x_O_4_ (where x = 0.00, 0.1, 0.2, 0.3, 0.4, and 0.5) ferrite nanoparticles and (**b**) variation of the lattice parameters with the concentration of Cr.

The X-ray diffraction (XRD) graphs of the collected samples confirmed the single-phase cubic spinel structures, as shown in Figure 1. The synthetic procedure of the samples and the synthetic conditions that took place are descripbed in detail in Section 4 (Materials and Methods).

### 2.2. Saturation Magnetization

**Figure 2:**
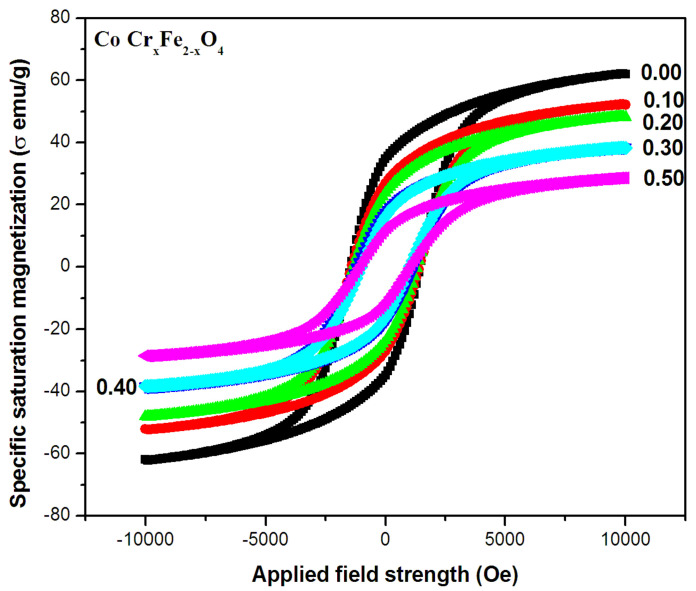
Room temperature hysteresis loops of CoCr_x_Fe_2_–_x_O_4_ (x = 0.00, 0.1, 0.2, 0.3, 0.4, and 0.5) nanoparticles.

Figure 2 depicts magnetic hysteresis loops for a series of CoCr_x_Fe_2−x_O_4_ samples with different values of x, ranging from 0.00 to 0.5 in 0.1 increments at ambient temperature. All samples show a soft ferrimagnetic character with low coercivities.

**Figure 3:**
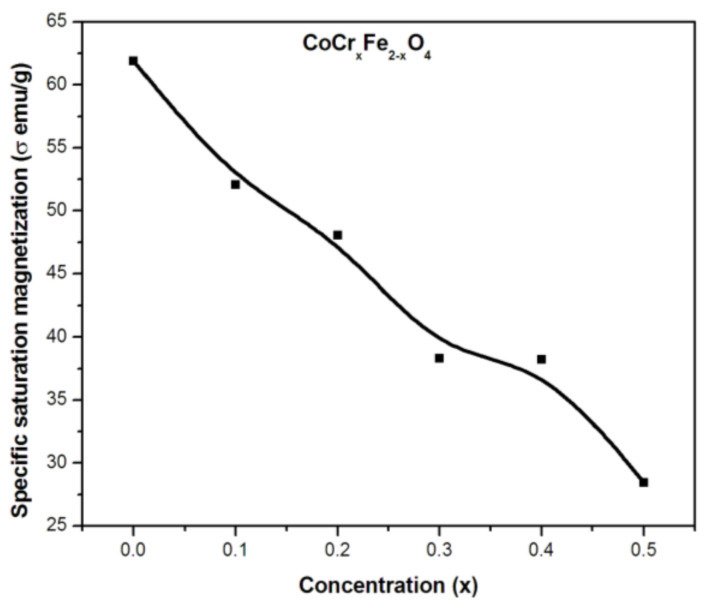
Variation in saturation magnetization with concentration (x) in CoCr_x_Fe_2−x_O_4_.

In addition, saturation magnetization (M_s_) and coercivity (H_c_) data were collected from the corresponding M-H curves for each sample. The change in M_s_ as a function of Cr ion substitution is shown in Figure 3. The observed changes in coercivities with changing substituent concentration are discussed in detail below.

The curves show that Ms decreases steadily and linearly with an increasing chromium (Cr) concentration in the CoCr_x_Fe_2−x_O_4_ system, with a significant decrease up to x = 0.5, followed by a substantial amplification up to x = 0.2. The observed variations in Ms as a function of Cr concentration can be explained by the hopping mechanism or superexchange associations of the constituent ions between the tetrahedral (A) and octahedral (B) sites in the spinel lattice of the cobalt ferrite. In a spinel lattice, the structure is often composed of two distinct types of ions or atoms, which are referred to as the A and B sublattices. These ions or atoms may have different magnetic properties, such as different electron configurations or spin values. In general, Neel examined three types of exchange interactions in ferrites between unpaired electrons of the ions located (i) at A-sites, occurring as a result of A–A interaction; (ii) at B-sites, occurring as a result of B–B interaction; and (iii) one at the A-site and another at the B-site, occurring as a result of A–B interaction. However, the superexchange interactions between A and B take preference over B–B and A–A. These interactions tend to have magnetic spins in opposite directions in each A and B site. As a result, the magnetic moment of the entire lattice is determined by the difference between the magnetic moments of the ions or atoms in the B sublattice (M_B_) and those in the A sublattice (M_A_). The type of ions or atoms present at both the A and B sublattices can have a significant influence on the exchange interactions within the spinel lattice.

The allocation of cations in the tetrahedral (A-side) sites, typically occupied by smaller cations, and the octahedral (B-side) sites, usually occupied by larger cations, in the spinel structure is indeed crucial when studying the compositional dependence of saturation magnetization in magnetic materials. Drawing on the work of past researchers can be immensely valuable in gaining a deeper understanding of the measurements and cation distribution in current research. Therefore, the cation distribution in CoFe_2_O_4_ can be represented by Equation (1):

(1) $$\left({Co}_{y}{Fe}_{1-y}\right)[{Co}_{1-y}{Fe}_{1+y}]O_{4}$$

Published works [1,2,3] focusing on cobalt ferrite systems indicated that the value of y is around 0.19. In the case of CoMn_x_Fe_2−x_O_4_ ferrite systems, it was expected that manganese (Mn) would exist in the Mn^3+^ form. This expectation arises from the fact that the sample was sintered in an air atmosphere, where Mn^2+^ ions can be oxidized into Mn^3+^ ions at temperatures between 200 and 450 °C [4,5]. On the basis of crystal field stabilization energy, Mn^3+^ ions are known to occupy the octahedral positions and replace Fe^3+^ ions [8,9,10,11]. With increasing manganese content, the B-sublattice magnetization falls constantly without impacting the A-sublattice magnetization, resulting in a drop in net magnetization. The observed variation in saturation magnetization in the aforementioned CoMn_x_Fe_2−x_O_4_ ferrite system was in contrast to the straightforward oxidation of available Mn into Mn^3+^ and the occupation of the same in B-sites; thus, an alternative mechanism would propose that a small amount of cobalt (z) would be transferred to the tetrahedral site, while manganese displaces iron ions at the octahedral sites. As a result, an amount of iron equivalent to cobalt would be transferred to the octahedral sites. This cobalt-to-A-site and iron-to-B-site transfer was thought to be proportionate to the manganese concentration (x). The cation distribution is portrayed by Equation (2):

(2) $$\left({Co}_{y+z}^{2+}{Fe}_{1-y-z}^{3+}\right)[{Co}_{1-y-z}^{2+}{Mn}_{x}^{3+}{Fe}_{1+y-x+z}^{3+}]O_{4}$$

The lack of non-magnetic ions at the tetrahedral sites promotes the major A–B exchange interactions between the cations present at both A- and B-sites. Thereby, the magnetic moment (M(x)) of the CoMn_x_Fe_2−x_O_4_ system was expressed as the difference of two sub lattice magnetizations, as given in Equation (3):

(3) $$\begin{array}{c} M\left(x\right)=\left(1-y-z\right)M_{Co}+\left(1+y-x+z\right)M_{Fe}+xM_{Mn}-\left(y+z\right)M_{Co}-(1-y-z)M_{Fe}\\ =3+4y+4z-x \end{array}$$

The magnetic moment values of the various cations, namely M_Co_ = 3 μ_B_, M_Fe_ = 5 μ_B_ and M_Mn_ = 4 μ_B_, taking part in Equation (3), were taken into consideration when calculating the value of M(x). With increasing manganese concentration, the magnetic moment M(x) showed an initial decrease for smaller values of z, followed by an increase for higher values of z. This calculation coincides with the experimentally observed variation in the magnetic moment up to x = 0.4. The observed decrease in saturation magnetization with increasing manganese concentration was attributed to a diminished superexchange interaction caused by the substitution of the tetrahedral Fe^3+^ ions by Co^2+^ ions [8,10].

In the case of CoCr_x_Fe_2−x_O_4_ systems, a steep decrease in saturation magnetization was expected owing to the auxiliary of Fe^3+^ (5 μB) ions by the paramagnetic Cr^3+^ (0 μB) ions. As there is a considerable difference in magnetic moments between substituting and displaced ions, it is expected that substituting paramagnetic chromium for iron will produce a rapid decrease in the magnetization by reducing the magnetic moment of the compound in each stage of iron ion replacement, as is represented in Equation (4):

(4) $$\left({Co}_{y}^{2+}{Cr}_{z}^{2+}{Fe}_{1-y-z}^{3+}\right)[{Co}_{1-y}^{2+}{Cr}_{x-z}^{2+}{Fe}_{1+y-x+z}^{3+}]O_{4}$$

Here, x represents the doped chromium concentration, y represents the cobalt concentration at the A-sites, and z represents any chromium concentration that may exist at the A-sites.

The allocation of cobalt and chromium ions among A- and B-sites was determined by considering the probable iron ion concentrations in those sites, as provided by Mössbauer spectra derived from previously reported studies [4,5,8,9,10,11]. The possibility of chromium occupying tetrahedral sites was also taken into account, since these materials might exhibit metastable cation distributions, especially as they are produced via the sol–gel autocombustion procedure within the size range of 20–40 nm. Based on fixed iron ion concentrations at the A- and B-sites, it appears that paramagnetic Cr^3+^ ions substitute for iron ions at both the A- and B-sites. This substitution results in a significant decrease in the magnetization of the B-sublattice and a less significant decrease in the magnetization of the A-sublattice. Additionally, this exchange process seems to induce the migration of cobalt from A-sites to B-sites to adjust the chromium occupancy in A-sites. Consequently, the net magnetization, which is the difference between these two sublattices, decreases more rapidly as the chromium concentration increases. As a result, the observed decrease in saturation magnetization in the Co-Cr ferrite system is consistent with this argument.

### 2.3. Maximum Magnetostriction

**Figure 4:**
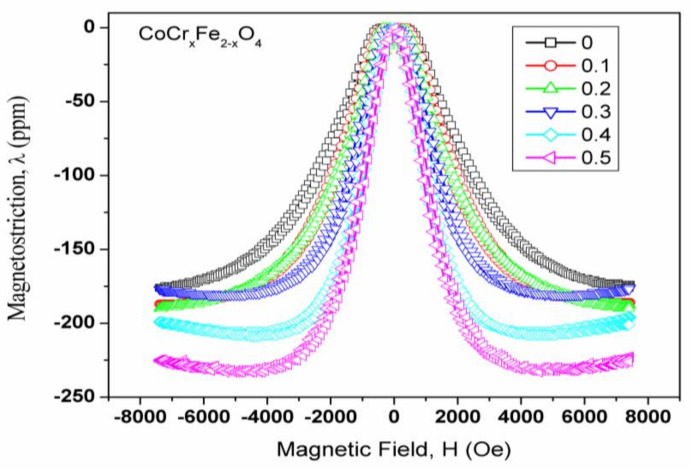
Magnetostriction curves as a function of magnetic field for CoCr_x_Fe_2−x_O_4_.

**Figure 5:**
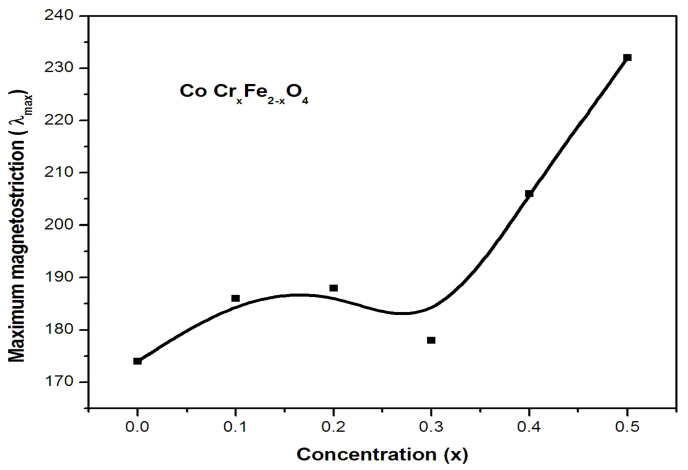
Variation in maximum magnetostriction with concentration (x) in CoCr_x_Fe_2−x_O_4_.

Figure 4 depicts magnetostriction curves with respect to applied magnetic field in a series of samples. These plots have been used to estimate the maximum magnetostriction and strain derivative values. Figure 5 depicts the differences in highest magnetostriction as a dependent of substituent concentration for ferrite samples. In the CoCr_x_Fe_2−x_O_4_ systems, the maximal magnetostriction increases moderately with chromium content at lower concentrations, but it increases significantly at higher concentration values.

The observed fluctuations can be explained using magnetostrictive contributions from various cations in the series of samples, their corresponding valence states, and site occupancy in a given ferrite composition [12,13,14,15]. The significant negative magnetostrictive contributions of Co^2+^ ions, as well as the large positive and sizeable negative magnetostrictive contributions of Fe^2+^ and Fe^3+^ ions, must be considered when explaining the magnetostrictive features of cobalt–ferrite-based systems [16,17,18].

Furthermore, the magnetostrictive contributions of the substituted cations, whether positive or negative, are likely to affect the resultant magnetostriction of the samples due to changes in the cation distribution caused by these substitutions. It should be noted that the magnetostrictive contribution at the tetrahedral site of a cation is known to be less than and opposite in nature to the octahedral site contribution for any given ion [19,20].

As shown previously in the CoMn_x_Fe_2−x_O_4_ systems, manganese resides in the Mn^3+^ state and displaces Fe^3+^ ions at the octahedral sites. The transfer of iron ions from tetrahedral to octahedral sites is thought to be proportional to the manganese concentration, but as manganese pushes iron ions out of octahedral sites, a small number of cobalt ions are compelled to migrate to that location. An increase in the population of Mn^3+^ ions with a positive magnetostrictive contribution and a decrease in the population of cobalt and Fe^3+^ ions with a negative magnetostrictive contribution at octahedral sites were, therefore, attributed to the observed decrease in magnetostriction in this system [16,18].

In CoCr_x_Fe_2−x_O_4_ systems, the saturation magnetization results show that as the concentration (x) increases, chromium ions replace iron ions at both the tetrahedral and octahedral sites in the crystal lattice. The observed increase in the maximum magnetostriction is primarily attributed to the higher presence of cobalt ions at B-sites. This presence of cobalt ions at B-sites induces a ‘trigonal distortion’ in the lattice structure, resulting in an increase in the maximal magnetostriction. This suggests that the arrangement of cobalt ions at B-sites has a significant effect on the material’s magnetostrictive behavior. The marginal increase in the maximum magnetostriction can be attributed to both the diluted population of cobalt ions and the concentrated population of chromium ions at the B-sites. This indicates that the interplay between cobalt and chromium ions at different sites in the crystal lattice plays a crucial role in the material’s magnetic behavior. A further rapid increase in maximum magnetostriction occurs as cobalt ions migrate to B-sites due to the corresponding occupation of chromium ions at the A-sites. As previously stated, the magnetostrictive contribution of tetrahedral cobalt ions and diamagnetic chromium is negligible.

### 2.4. Strain Derivative (dλ/dH)

**Figure 6:**
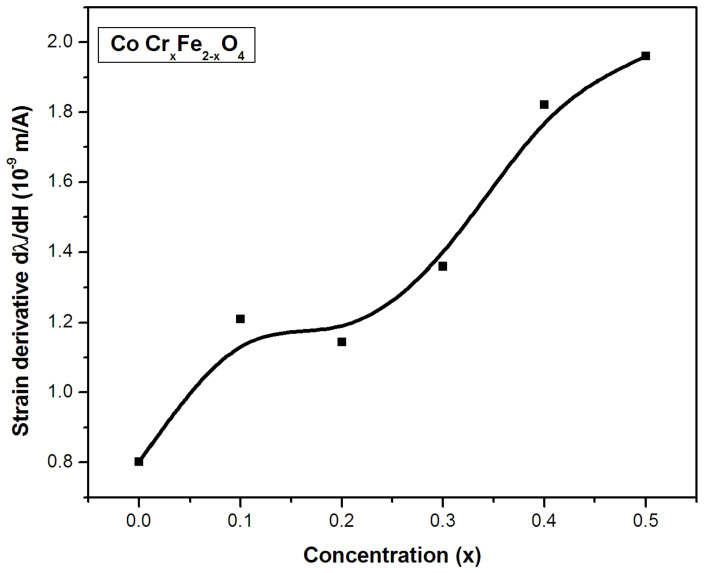
Variation in strain derivative with concentration (x) in CoCr_x_Fe_2−x_O_4_.

Figure 6 depicts the variations in the magnetostrictive strain derivative ($\frac{d\lambda }{dH}$) as a function of the substituent concentration for a series of ferrite samples. The strain derivative in the Co-Cr ferrite system increased with the chromium content across the whole range of substitutions in the CoCr_x_Fe_2−x_O_4_ systems. Therefore, this implies that the chromium-substituted system exhibited an increasing trend in the range of concentration from x = 0 to 0.1, and, thereafter, a decrease is marked at higher concentrations of chromium.

As the strain derivative depends on both the magnetostriction and anisotropy of the material, the observed variations in the strain derivative with substituent concentration can be explained using magnetic anisotropy and the magnetostrictive contributions of the cations present in the sample [21]. A thermodynamic relationship [22] exists for minor variations in the magnetic field (H) and applied stress (σ), as given in Equation (5).

(5) $${\left(\frac{d\lambda }{dH}\right)}_{\sigma }={\left(\frac{dB}{d\sigma }\right)}_{H}$$

The strain derivative is related to the ratio of saturation magnetostriction (λ_max_) to cubic anisotropic constant (K) [21], as demonstrated by the single-ion anisotropy-based investigation [23]. The magnetostriction-to-anisotropy ratio is, indeed, an important factor in understanding and controlling the behavior of magnetic materials in response to stress, which may be expressed by Equations (6) and (7), as shown below.

$$\frac{dB}{d\sigma }=\left(\frac{d\lambda }{dH}\right)=(\frac{d\lambda }{dM})(\frac{dM}{dH})$$

(6) $$\frac{dB}{d\sigma }=\frac{2\mu_{o}\lambda_{s}M}{NK}$$

This implies that

(7) $$\frac{d\lambda }{dH}\propto \frac{\lambda }{K}$$

The magnetostriction was experimentally determined, whereas the anisotropy constant was calculated from the relationship mentioned in Equation (8) (Globus–Duplex):

(8) $$\mu_{i}\propto \frac{M_{s}^{2}D}{K}$$

where μ_i_ is the initial permeability, M_s_ is the saturation magnetization, D is the grain size, and K is the anisotropy constant.

The orbital degeneracy of an ion is an important factor in determining its magnetic anisotropy. In the context of transition metal ions, which are known for their important roles in magnetism, the relationship between orbital degeneracy and magnetic anisotropy is significant. When an ion is in a non-degenerate orbital state, it means that all of its electrons occupy distinct, non-degenerate orbitals. In this case, there are no multiple, energetically equivalent orbitals available for electrons to occupy. As a result, magnetic anisotropy cannot be anticipated.

Earlier reported studies suggest that the magnetic anisotropy in cobalt ferrites is proportional to the cobalt content [24,25]. Furthermore, octahedral sites of Co^2+^ ions are acknowledged to contribute more anisotropy to the cobalt ferrite complex via trigonal deformation than cobalt ions at tetrahedral sites [26]. The minor degeneracy connected with Cr^3+^ ions over Fe^3+^ ions at octahedral locations leads to decreased anisotropy and increased λ/K at lower chromium ion concentrations. In the reported cobalt–manganese ferrite systems, where manganese replaces iron ions, an increase in the anisotropy constant is mentioned [27,28,29]. In addition, at higher concentrations of manganese, the observed reduction in the number of cobalt ions at octahedral sites can be the reason for a decrease in the magnetic anisotropy contribution, resulting in an increase in the value of λ/K [30,31]. However, in CoCr_x_Fe_2−x_O_4_ systems, we assume that the iron ions have been replaced by chromium ions at both A- and B-sites, respectively. Similar arguments were made earlier when discussing variations in magnetization. In light of these arguments, the contributions of cobalt ions at tetrahedral sites and the non-degenerate paramagnetic chromium ions at octahedral sites toward magnetostriction and magnetic anisotropy in the systems are considered insignificant. This leads to a continuous increase in the strain derivative with substituent content. Moreover, the observed continuous increase in the strain derivative with an increase in the substituent concentration implies that the magnetic anisotropy is reduced to a greater degree than magnetostriction, resulting in larger strain derivative values. However, the observed decrease in the strain derivative when the chromium concentration is equal to x = 0.2 may be attributed to the presence of a separate second phase, such as chromium oxide. In low fields, the presence of any non-magnetic second phase is known to impede magnetization processes and lower the strain derivative. Nevertheless, the most interesting aspect of the chromium-substituted system lies in exhibiting maximum values of both maximum magnetostriction and strain derivative between x = 0.2 and 0.5. As a result of the combination of good magnetic characteristics and magnetostriction values, these materials can be suitable candidates for magnetostrictive applications.

### 2.5. Coercivity

**Figure 7:**
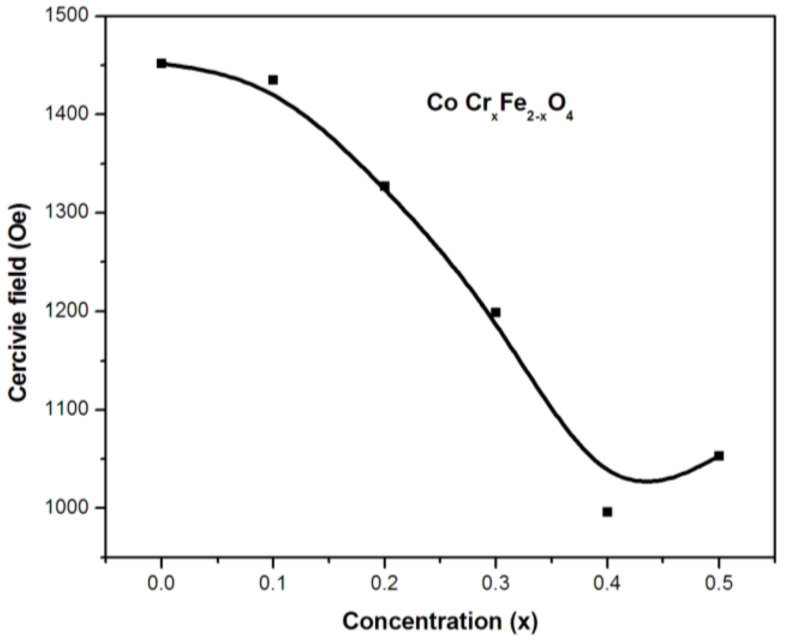
Variation in coercivity with concentration (x) in CoCr_x_Fe_2−x_O_4_.

Figure 7 shows the relationship between coercivity and the chromium (Cr) concentration in a series of samples within the CoCr_x_Fe_2−x_O_4_ system. It is noted that there is an exception at the x = 0.5 concentration, where a progressive decline was observed. On the other hand, the magnitude of the gradient from the lowest chromium-substituted cobalt ferrite (x = 0.0) to the basic cobalt ferrite (x = 0.0) is relatively constant, suggesting that the anisotropic and microstructural changes responsible for the coercivity variations in the substituted ferrite systems are similar.

Magnetocrystalline anisotropy, lattice defects, dislocations, internal stresses, particle size, and secondary phases are all known to influence coercivity in ferrites [32,33,34]. In this context, the rise of cobalt ions at tetrahedral locations is responsible for a decrease in coercivity in the synthesized CoCr_x_Fe_2−x_O_4_ ferrites. This indicates that the specific arrangement of cobalt ions within the crystal lattice has a significant impact on the magnetic properties of these materials, leading to a reduction in coercivity. According to magnetization experiments, the additional chromium ions replace the iron ions at both the tetrahedral and octahedral positions [35,36,37,38,39]. In the concentration range of x = 0.1 to 0.2, the substantial fall in coercivity and a comparable increase in the strain derivative confirm our theory about cobalt’s migration to A-sites.

## 3. Conclusions

In summary, this study explored the impact of chromium substitution on the magnetic and magnetostrictive properties of cobalt ferrite nanoparticles synthesized using the sol–gel autocombustion process [40]. In general, substituting one metal ion for another can affect the overall properties of the material. Thus, the observed linear drop in magnetization values up to x = 0.1 was attributed to the superexchange interactions between the tetrahedral (A) and octahedral (B) sites in the spinel lattice. A decrease in coercivity was observed as the chromium content increased, and this decrease is attributed to an increase in cobalt ions occupying the tetrahedral positions in the lattice. Moreover, the decline in coercivity is related to the decreased contribution of the anisotropy of cobalt ions, which may be caused by chromium ion hopping at tetrahedral and octahedral sites. Nevertheless, the experimental decrease in the strain derivative with chromium concentrations up to x = 0.2 was attributed to the presence of a separate second phase, namely chromium oxide. In general, the presence of any non-magnetic secondary phase is known to obstruct magnetization processes and reduce the strain derivative in lower fields. However, the most interesting feature of this chromium-replaced system lies in displaying the maximum values of both the maximum magnetostriction and strain derivative between x = 0.2 and 0.5, combined with good magnetic parameters. Thus, these materials represent suitable candidates for future magnetostrictive applications.

## 4. Materials and Methods

The sol–gel autocombustion method is a commonly used technique for synthesizing nanoparticles, including ferrite nanoparticles. In this method, a sol (a stable colloidal suspension of nanoparticles in a liquid) is prepared from precursor chemicals, and then the sol is subjected to combustion to form the desired nanoparticles. Ferrite nanoparticles with the chemical formula CoCr_x_Fe_2−x_O_4_ were synthesized in this work, and x can take on various values (0.00, 0.1, 0.2, 0.3, 0.4, and 0.5). The starting materials used in this work included analytical reagent grade cobalt nitrate, iron nitrate, chromium nitrate, citric acid, and ammonia. All of the chemicals were acquired from Hi-media in Mumbai, India, and were used without further modification.

The necessary amounts of the metal nitrate salts and citric acid solutions were prepared separately using small amounts of deionized water, and then they were combined in a 1:1 molar ratio to create an aqueous solution, a process that maximizes the molecular mixing of the constituents. The pH of the solution was adjusted to seven using ammonia. This was carried out to create the ideal conditions for the subsequent stages of the process. The fluid was heated to 80 °C while being agitated. This step is crucial for the formation of the dry gel. The container was heated up to 110 °C at this point, ready to catch fire at any moment. The dried gel was ignited, and it burned in a self-replicating combustion pattern. This process consumes the gel until only ash flakes remain. After combustion, the ash flakes were carefully gathered and collapsed using a glass rod or spatula. This resulted in the formation of fluffy slack nano-powders. The nano-powders were subsequently calcined at 900 °C for 2 h in an air medium.

The assembly of ferrite pellets and toroids using nanoparticles in the size range of 40–45 nm, based on XRD data, was accomplished using standard ceramic techniques [10]. The obtained ferrite nanoparticles were gently milled in methanol for a few hours, air-dried, and then granulated with polyvinyl alcohol (PVA-5%) as a binder. PVA is often used as a binder in various applications, including forming solid shapes from fine powders. At a pressure of 150 MPa, the granulated powder was pressed into pellets and toroids. The resulting compacts of each ferrite system were sintered in an air environment at 1200 °C for 4 h.

After reaching the soaking temperature, the samples were cooled down gradually at a rate of approximately 200 °C per hour. After turning off the furnace, the samples were allowed to cool naturally overnight.

The sintered pellets and toroids were then subjected to various tests. Archimedes’ method was used to calculate the bulk density of the sintered ferrite nanoparticles. The microstructure was studied using scanning electron microscopy (SEM), as well as grain sizes, which were calculated from the collected micrographs using the linear intercept approach that we previously published. Also, magnetic measurements on toroidal samples were carried out using an HP LF4192A impedance analyzer with a frequency range of 1 kHz to 13 MHz.

## Data Availability

Any additional data in support of the findings of this study are available from the corresponding author upon reasonable request.
